# Comparative genomic, proteomic and exoproteomic analyses of three *Pseudomonas* strains reveals novel insights into the phosphorus scavenging capabilities of soil bacteria

**DOI:** 10.1111/1462-2920.13390

**Published:** 2016-07-07

**Authors:** Ian D. E. A. Lidbury, Andrew R. J. Murphy, David J. Scanlan, Gary D. Bending, Alexandra M. E. Jones, Jonathan D. Moore, Andrew Goodall, John P. Hammond, Elizabeth M. H. Wellington

**Affiliations:** ^1^ School of Life SciencesUniversity of WarwickGibbet Hill RoadCoventryWest MidlandsCV4 7ALUK; ^2^ The Genome Analysis Centre, Norwich Research ParkNorwichNR4 7UHUK; ^3^ School of Agriculture, Policy, and DevelopmentUniversity of ReadingEarley GateWhiteknightsReadingRG6 6ARUK; ^4^ Southern Cross Plant Science, Southern Cross UniversityLismoreNSW 2480Australia

## Abstract

Bacteria that inhabit the rhizosphere of agricultural crops can have a beneficial effect on crop growth. One such mechanism is the microbial‐driven solubilization and remineralization of complex forms of phosphorus (P). It is known that bacteria secrete various phosphatases in response to low P conditions. However, our understanding of their global proteomic response to P stress is limited. Here, exoproteomic analysis of *Pseudomonas putida* BIRD‐1 (BIRD‐1), *Pseudomonas fluorescens* SBW25 and *Pseudomonas stutzeri* DSM4166 was performed in unison with whole‐cell proteomic analysis of BIRD‐1 grown under phosphate (Pi) replete and Pi deplete conditions. Comparative exoproteomics revealed marked heterogeneity in the exoproteomes of each *Pseudomonas* strain in response to Pi depletion. In addition to well‐characterized members of the PHO regulon such as alkaline phosphatases, several proteins, previously not associated with the response to Pi depletion, were also identified. These included putative nucleases, phosphotriesterases, putative phosphonate transporters and outer membrane proteins. Moreover, in BIRD‐1, mutagenesis of the master regulator, *phoBR*, led us to confirm the addition of several novel PHO‐dependent proteins. Our data expands knowledge of the *Pseudomonas* PHO regulon, including species that are frequently used as bioinoculants, opening up the potential for more efficient and complete use of soil complexed P.

## Introduction

Phosphorus (P) is an essential macroelement for all living biota. In soil, microorganisms and plants compete for P. It is therefore essential that a sufficient amount of P is available for agricultural crops to sustain their yields. Plants acquire P as inorganic orthophosphate (Pi) from the soil solution (Vance *et al*., 2003; White and Hammond, 2008). The concentration of Pi in the soil solution is controlled by chemical and biological processes which fix and release Pi through complex interactions between the soil, soil microorganisms and plant roots (Richardson *et al*., 2009; Shen *et al*., 2011). To overcome these limitations in agricultural systems, inorganic fertilizers, derived from non‐renewable Pi rocks, are supplied to crops and pastures (Vance *et al*., 2003; López‐Arredondo *et al*., 2014). Over 85% of mined P is used in food production (Heffer *et al*., 2006) and consumption of this non‐renewable resource could lead to a peak P scenario (akin to peak oil; Raven, 2008; Cordell *et al*., 2009). It is, therefore, likely that there will be increasing pressures on Pi fertilizer availability and, consequently, cost in the future. These pressures will be exacerbated by increasing demand on food production systems as the human population increases and by fluctuation in oil prices (Cordell *et al*., 2009). Inappropriate use of inorganic Pi fertilizers can also perturb the nutrient balance of natural ecosystems and reduce biodiversity (White and Hammond, 2008, 2009). Hence, it is desirable to increase the efficiency by which plants can access the many forms of unavailable P that reside within soil, thus, reducing the requirements for Pi fertilizer application (Richardson *et al*., 2009; Stutter *et al*., 2012).

Plant‐growth promoting rhizobacteria (PGPR) are bacteria that can enhance crop yields through a variety of mechanisms, including P mobilization, and their identification has led to the notion that bacteria are an integral part of the plant‐root interface (rhizosphere) (Lugtenberg and Kamilova, 2009; Miller *et al*., 2010). One advantage of utilizing P‐liberating bacteria in agricultural systems is that they can have synergistic beneficial effects, for example, pathogen suppression (Vassilev *et al*., 2006). The majority of research into P mobilization by PGPR has focused on the solubilization of inorganic P through acidification of the surrounding soil via the release of organic acids, namely gluconic acid (Rodrı'guez and Fraga, 1999; Miller *et al*., 2010; Oteino *et al*., 2015). The genus *Pseudomonas* represents one soil bacterial group that is frequently associated with plant‐growth promotion, including P solubilization (Miller *et al*., 2010). Various *Pseudomonas* strains can also degrade organic P compounds, such as phytate, phosphonates and phosphites (Ternan and Quinn, 1998; White and Metcalf, 2004, 2007). Three *Pseudomonas* strains*, Pseudomonas putida* BIRD‐1, *Pseudomonas fluorescens* SBW25 and *Pseudomonas stutzeri* DSM4166 (hereafter, BIRD‐1, SBW25 and DSM4166 respectively) are three examples of PGPR (Naseby *et al*., 2001; Hass and Keel, 2003; Preston, 2004; Yu *et al*., 2011; Roca *et al*., 2013). SBW25 inhabits the rhizosphere of Pea plants and is antagonistic towards the pathogen *Pythium ultimum* (Naseby *et al*., 2001), whereas DSM4166, an ‘unusual’ nitrogen‐fixing bacterium, was isolated from a cultivar of *Sorghum nutans* (Yu *et al*., 2011). BIRD‐1, a P‐solubilising bacterium, has been previously utilized as a bioinoculant, since it can significantly improve the germination rates, growth and yields of various agricultural crops (Roca *et al*., 2013). BIRD‐1 can remineralize Pi from the plant P‐storage compound phytate, a major source of organic P in some soils (up to 50%) (Stutter *et al*., 2012). Furthermore, when BIRD‐1 was used as an inoculant, phosphatase activity was greater in the rhizosphere compared with bulk soil (Roca *et al*., 2013). Although *Pseudomonas* have been implicated in plant‐growth promotion, partially attributed to their effect on P mobilization, the precise mechanisms behind this process remain largely unknown.

The majority of Bacteria studied can undergo a physiological response to Pi depletion controlled by a two‐component regulatory system (PhoBR) encoded by *phoBR*. PhoBR regulates a large set of genes (the PHO regulon) in response to low P concentrations (Baek and Lee, 2006; Monds *et al*., 2006; Su *et al*., 2007). The majority of studies have focused on specific functions/mechanisms that are regulated by PhoBR, for example, the Pi specific transport (Pst) system, motility and swarming, and expression of alkaline phosphatases (APases), acid phosphatases or secondary metabolites (Rittmann *et al*., 2005; Monds *et al*., 2006; Sola‐Landa *et al*., 2008; Furtwängler *et al*., 2010; Zavaleta‐Pastor *et al*., 2010). Fewer studies have experimentally confirmed the global PHO regulon using recently developed ‘omics’ techniques. Microarrays were employed to identify genes regulated at the transcriptional level in *Pseudomonas aeruginosa* (Bains *et al*., 2012), *E. coli* (Baek and Lee, 2006) and *Synechococcus* sp. WH8102 (Tetu *et al*., 2009; Ostrowski *et al*., 2010) while traditional 2D‐gel electrophoresis was performed to make a qualitative assessment of the *Bacillus subtilis* proteome in response to low Pi (Antelmann *et al*., 2000). These studies identified a number of genes/proteins involved in Pi scavenging underlining the importance of performing ‘omics’ to study Pi acquisition. In *Pseudomonas*, it has been shown that phosphate binding proteins (PBPs), APases, and virulence factors are associated with phosphate‐stress and regulated by PhoBR (Monds *et al*., 2006; Bains *et al*., 2012; Putker *et al*., 2013: Santos‐Beneit, 2015).

Exoproteomics captures the extracellular protein fraction that results from active secretion, cell lysis or leakage during cell division (Armengaud *et al*., 2012; Ebner *et al*., 2016). Removing the cellular fraction prior to protein extraction can help to identify exoproteins that are involved in the interaction of microorganisms with their environment (Christie‐Oleza *et al*., 2012, 2015). The majority of exoproteins detected are associated with nutrient acquisition, motility, cell attachment, defence, communication as well as antagonism (Christie‐Oleza *et al*., 2012). Therefore, exoproteomics is the ideal method of choice to study the bacterial mechanisms for scavenging extracellular P. Due to the complexity and technical challenges associated with metaproteomics (Muth *et al*., 2015), this study aimed to identify Pi‐responsive proteins that can be used as markers for future studies investigating Pi mobilization within the rhizosphere. Therefore, exoproteomic analyses were performed on three plant‐associated *Pseudomonas* strains grown under Pi‐deplete conditions (50 μM). We hypothesized that *Pseudomonas* strains harbour a number of common Pi‐scavenging enzymes that would be expressed during Pi depletion. In reality, clear evidence for intra‐genus‐level heterogeneity in their exoproteomes was observed and a number of novel PHO‐regulon members specifically linked with the PHO regulon in *Pseudomonas putida* BIRD‐1 were also determined.

## Results

### Effects of P‐limitation on the growth of *Pseudomonas* strains

**Figure 1:**
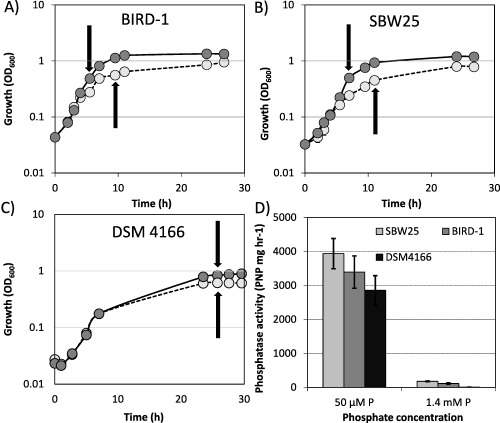
A–C. Growth of the three *Pseudomonas* strains under either Pi replete (1.4 mM) or Pi deplete (50 μM) growth conditions. Arrows denote sampling points for exoproteomics. D. During the growth experiments, alkaline phosphatase activity was quantified as a proxy for determining the activation of the PHO regulon. The values shown represent a given time point when the maximal alkaline phosphatase activity detected for each strain was obtained. Results presented are the mean of triplicate cultures. Error bars denote standard deviation.

We investigated the effect of Pi stress on three strains, DSM4166, SBW25) and BIRD‐1 by comparing growth under Pi‐replete (1.4 mM) or Pi‐deplete (50 µM) conditions (*n* = 3). Both the growth rates and growth yields of all three *Pseudomonas* strains showed a significant decrease (*t*‐test score, *P* < 0.01) under Pi‐deplete growth conditions (Fig. 1A–C). As expected, Pi‐deplete cultures of all three strains demonstrated a significant increase in the level of APase activity (Fig. 1D) due to the induction of the PHO regulon (Monds *et al*., 2006; Putker *et al*., 2013).

### General characteristics of the exoproteomes of the three *Pseudomonas* species

**Figure 2:**
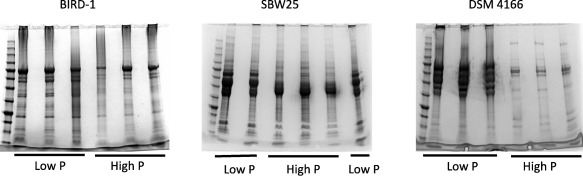
A qualitative assessment, using 1D‐SDS PAGE, of the exoproteomes of all three *Pseudomonas* strains examined prior to HPLC 2D‐MS/MS. Each gel lane represents 20 ml culture supernatant. For both Pi deplete and Pi replete growth conditions, three biological replicates were performed.

**Figure 3:**
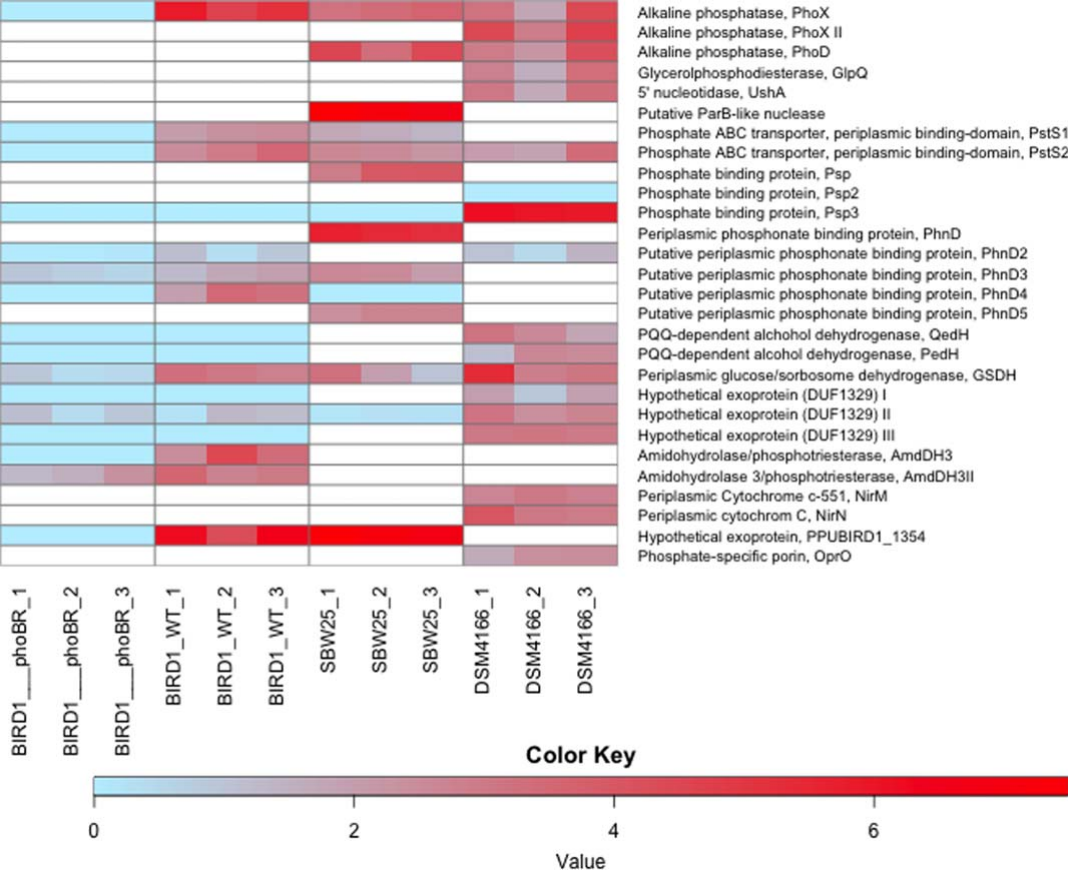
Protein expression analyses in response to Pi depletion of the three *Pseudomonas* exoproteomes in addition to the exoproteome of the *phoBR* mutant of *P. putida* BIRD‐1. White spaces represent the absence of genes encoding the corresponding proteins from their genomes. Each individual biological replicate is displayed. The colour key represents Log2 transformations of protein fold change.

**Figure 4:**
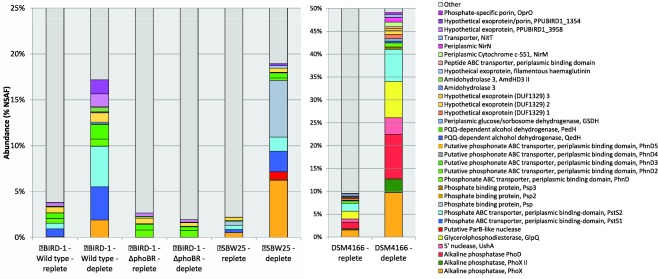
The relative abundance of Pi scavenging proteins detected in the exoproteomes of the three *Pseudomonas* strains and the *phoBR* mutant grown in both Pi replete and Pi deplete growth conditions. The normalized spectral abundance factor (NSAF) was calculated using Scaffold 4. Values displayed are the mean of triplicate cultures.

Based on qualitative 1D SDS‐PAGE analysis, there was visible evidence for heterogeneity in the profiles of *Pseudomonas* exoproteomes in response to Pi‐depletion (Fig. 2). Samples were subsequently processed for peptide identification using LC‐MS/MS. Proteins were considered present based on a minimum of at least two unique peptides. Exoproteins were identified by the presence of a signal peptide sequence (IMG/JGI). Proteins detected in the exoproteome that did not possess a signal peptide sequence were further analysed using SecretomeP and LipaseP to determine if they are secreted in a non‐classical manner (Christie‐Oleza and Armengaud, 2010; Christie‐Oleza *et al*., 2015). In Pi‐deplete cultures, ‘predicted’ exoproteins comprised 89.9%, 68.3% or 97.6% of the top‐60 most abundant proteins detected in the exoproteomes of BIRD‐1, SBW25 and DSM4166 respectively (Supporting Information Fig. S1), while the remaining fraction was comprised of cytoplasmic proteins. In BIRD‐1, SBW25 and DSM4166 29, 52 and 54 proteins were significantly enriched (*t*‐test, *P* value ≤ 0.05, fold‐change (log2) ≥ 1.5) in response to Pi‐depletion respectively (Supporting Information Tables S1–S3). In all three *Pseudomonas* exoproteomes a suite of previously characterized PHO‐dependent proteins (Santos‐Beneit, 2015) were enriched in their exoproteomes during growth under Pi depletion. These included the high affinity periplasmic substrate binding protein (SBP) subunits of the high affinity Pi transporter (PstS) and phosphonate transporter (PhnD), APases (PhoX, PhoD), 5'‐nucleotidase (UshA) and glycerolphosphodiesterase (GlpQ) (Figs. 3 and 4 and Supporting Information Fig. S2). However, the genomic content (Table 1) and thus exoproteomic response to Pi depletion varied between the three *Pseudomonas* strains (Fig. 3), revealing significant inter‐genus level heterogeneity. We should point out though that the exoproteome of DSM4166 was harvested after a longer period (DSM4166, 25 h; BIRD‐1 & SBW25, 7–11 h respectively) of Pi stress (Fig. 1) and as a result a higher percentage of its exoproteome was associated with Pi‐scavenging compared with either BIRD‐1 or SBW25 (Fig. 4).

**Table 1:** Comparative genomic analysis of selected proteins involved in the recycling of P among *Pseudomonas* isolates.

	*P. aeruginosa* PAO1	*P. fluorescens* A506	*P. fluorescens* F113	*P. fluorescens* Pf0‐1	*P. fluorescens* SBW25	*P. putida* BIRD‐1	*P. putida* GB‐1	*P. putida* KT2440	*P. putida* W619	*P. stutzeri* A1501	*P. stutzeri* DSM4166	*P. stutzeri* DSM10701	*P. syringae* CC1557	*P. syringae* pv. actinidia ICMP 9617	*P. syringae* B728a	*P. syringae* D C3000
**P_i_ transport**																
PstS1		♦			♦	♦	♦	♦								
PstS2	♦	♦	♦	♦	♦	♦	♦	♦	♦	♦	♦	♦	♦	♦	♦	♦
LapA/LapB	♦			♦												
Psp			♦		♦											
Psp2										♦	♦					
Psp3	♦	♦	♦	♦	♦	♦	♦	♦	♦	♦	♦	♦	♦	♦	♦	♦
Psp4																
**P_o_ scavenging**																
PhnF‐M	♦				♦								♦	♦	♦	♦
PhnXW	♦	♦		♦	♦	♦	♦	♦	♦							
PhnWAY																
PhnCDE	♦				♦								♦	♦	♦	♦
PhnC_2_D_2_E_2_	♦		♦	♦		♦	♦	♦	♦	♦	♦	♦	♦	♦	♦	♦
PhnD3		♦	♦	♦	♦	♦	♦	♦	♦				♦	♦	♦	♦
PhnD4		♦		♦	♦	♦	♦	♦	♦							
PhnD5			♦		♦								♦	♦	♦	♦
PalA																
PhoA	♦															
PhoX	♦	♦	♦	♦	♦	♦	♦	♦	♦	♦	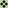	♦	♦	♦	♦	♦
PhoD	♦	♦	♦	♦	♦					♦	♦		♦	♦	♦	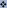
GlpQ	♦									♦	♦	♦				
Phytase		♦	♦		♦					♦	♦	♦	♦	♦		♦
UshA						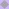		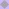			♦					
**Lipid renovation**																
PlcP	♦	♦	♦	♦	♦	♦	♦	♦	♦				♦	♦	♦	♦
DagK	♦	♦	♦	♦	♦	♦	♦	♦	♦				♦	♦	♦	♦
OlsA	♦	♦	♦	♦	♦	♦	♦	♦	♦	♦	♦	♦	♦	♦	♦	♦
OlsB	♦	♦	♦	♦	♦	♦	♦	♦	♦	♦	♦	♦	♦	♦	♦	♦
OlsF																
SqdBCD																
Cfa	♦	♦	♦	♦	♦	♦	♦	♦	♦	♦	♦	♦	♦		♦	
BtaAB																

BLASTP was performed using the IMG/JGI database on selected strains whose genome was marked as ‘finished’. Multiple diamonds indicate two or more homologs.

Abbreviations are the same as in Figure 2 as well as: LapA/B, low molecular weight phosphatase; PhoT5, Flavobacterial putative Pi‐binding protein form V; PhnF‐M, C‐P lyase; PhnXW, phosphonatase; PhnWAY, alternative 2‐aminoethylphosphonatase; PhnCDE, phosphonate transporter I, PhnC_2_D_2_E_2_, putative phosphonate transporter II; PalA, phosphonopyruvate hydrolase; PhoA, alkaline phosphatase; PlcP, intracellular phospholipid phosphodiesterase; DagK, diacylglycerol kinase; OlsA, Lyso‐ornithine lipid:acyl‐ACP O‐acyltransferase; OlsB, Ornithine:acyl‐ACP N‐acyltransferase; OlsF, bifunctional ornithine acyltransferase; SqdBCD, sulfolipid biosynthesis; Cfa, cyclo‐propane fatty acid synthase; BtaAB, diacylglycerol trimethylhomoserine biosynthesis.

### Pseudomonads show heterogeneity in their resource allocation towards organic P scavenging

Bacteria possess a number of different APases (PhoA, PhoD, PhoX) with different phosphomonoesterase and phosphodiesterase activities (Brickman and Beckwith, 1975; Scott and Wu, 2005). Again, there is genomic and thus exoproteomic variation between the three *Pseudomonas* strains with respect to the catabolism of organic P (Fig. 3 and Table 1). For example, BIRD‐1 possesses PhoX but lacks PhoD whereas DSM4166 and SBW25 possess both exoenzymes. Furthermore, PhoD was the second most abundant protein in DSM4166 while it was ranked 355th in the exoproteome of SBW25. In addition, DSM4166 also harbours a distinctive PhoX homolog that is duly expressed under Pi depletion (Fig. 4). DSM4166 also expressed homologs of GlpQ (Larson *et al*., 1983) and UshA (Zalkin and Nygaard, 1996; Rittmann *et al*., 2005; Pinchuk *et al*., 2008) while BIRD‐1 and SBW25 do not possess either of these exoenzymes. BIRD‐1 did possess a gene encoding a predicted exoprotein containing the same domains as UshA (Pfam00149 – metallophos; Pfam02872 – 5_nucleotid_C), but did not secrete this protein in response to low Pi.

### Pseudomonads harbour a number of phosphate binding proteins (PBPs) that are enriched in their exoproteome in response to Pi‐depletion

**Figure 5:**
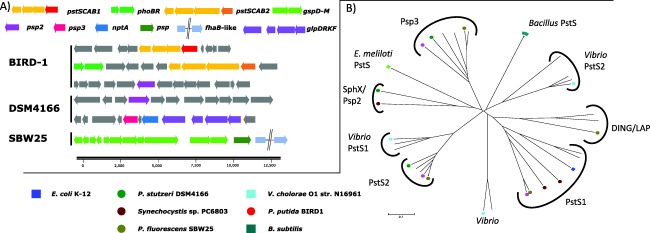
Genomic analyses of the Pi binding proteins found in the three *Pseudomonas* strains (A) The genetic neighbourhood profiles of the different Pi binding proteins located in the three *Pseudomonas* strains (B) The diversity of proteins that contain the Pfam12849 domain using a number of genome‐sequenced soil bacteria with the inclusion of characterized Pi binding proteins. Abbreviations; *pstSCAB*1/2, Pi‐specific ABC transporter; *psp*, DING‐family Pi binding protein; *psp2/3*, uncharacterized Pi binding protein; *nptA*, NA^+^/Pi co‐transporter; *glp*D, glycerol 3‐phosphate dehydrogenase; *glpR*, transcriptional regulator; glycerol kinase; *glpK*, glycerol uptake facilitator; *fhaB*‐like, putative filamentous haemagglutinin; *phoBR*, two component regulator; *gsp*, type II secretion system.

In total, the three *Pseudomonas* strains increased the secretion of four different Pi binding proteins (PBP) containing the Pfam01249 domain, in response to Pi‐depletion (Figs. 3 and 4). All PBPs found in the genomes of the three *Pseudomonas* strains contain the key residues associated with Pi binding (Supporting Information Fig. S3) (Berna *et al*., 2008; Liebschner *et al*., 2009). DSM4166 has a single operon encoding Pst (*pstSCAB*), whereas BIRD‐1 and SBW25 have two operons encoding two separate Pst systems (Fig. 5A). All PstS homologs were secreted in large quantities during Pi depletion (Fig. 4). The two operons were named *pst1* and *pst2*. PstS1 is closely related to PstS found in *E. coli* (Wanner, 1990) and *Synechocystis* sp. PCC6803 (herein, *Synechocystis*) (Pitt *et al*., 2010), whereas PstS2 is phylogenetically distinct and closely related to *Vibrio* PstS (Pratt *et al*., 2010) (Fig. 5B). Interestingly, *pstSCAB1* is not present in all Pseudomonads whereas *pstSCAB2* is (Table 1).

In SBW25, another PBP (encoded by PFLU2427), hereafter referred to as Psp, was heavily secreted in Pi‐deplete cultures (Fig. 4) alongside a large hypothetical exoprotein (encoded by PFLU2428) that contains a domain related to an adhesion virulence factor (Inatsuka *et al*., 2005). All *Pseudomonas* screened encode Psp3. However, only DSM4166 appeared to secrete this exoprotein (PSTAA_2217) in response to Pi stress. Psp3 contains an outer membrane protein A (OmpA) domain implying that this protein may be located in the outer membrane.

### Acquisition of phosphonates in phosphate‐depleted *Pseudomonas* cells

**Figure 6:**
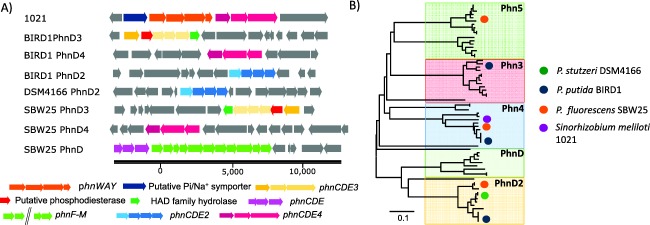
Genomic and proteomic analyses of the phosphonate binding proteins found in the three *Pseudomonas* strains (A) The genetic neighbourhood profiles of the different phosphonate binding proteins located in the three *Pseudomonas* strains (BIRD‐1, SBW25, DSM4166) as well as *Sinorhizobium meliloti* 1021 (1021). (B) Neighbour‐joining phylogenetic analysis of the different phosphonate binding proteins detected in the *Pseudomonas* strains outlined in Table 1 with the addition of various *Burkholderia* and *Flavobacteria* strains. Bootstrap values (500 runs) have been omitted for clarity. IMG accession numbers have been included as a reference. Abbreviations: *phnWAY*, alternative 2‐aminoethylphosphonate degradation pathway; *phnDCE1/2/3/4*, phosphonate ABC transporter; *phnF‐M*, C‐P lyase.

To date, PhnD, which is located in an operon with genes encoding the promiscuous C‐P lyase (PhnF‐M), is the only characterized phosphonate transporter (Baker *et al*., 1998). A number of putative SBPs responsible for phosphonate transport were identified in the *Pseudomonas* strains genomes (Fig. 6A) and detected in their exoproteomes, with almost all showing a positive response to Pi‐depletion (Supporting Information Fig. S4). Phylogenetic analysis categorized these homologs into five groups, hereafter referred to as PhnD, PhnD2, PhnD3, PhnD4 and PhnD5. However, none of the three *Pseudomonas* strains possesses all five homologs in their genomes (Fig. 6B and Table 1). PhnD and PhnD2 both contain the Pfam012974 domain (phosphonate‐binding domain) and these two homologs are mutually exclusive with one another among the genomes of *Pseudomonas* strains. Only SBW25 possesses and secreted PhnD, a known PHO‐regulon member (Baker *et al*., 1998), during Pi‐depletion (Fig. 6). BIRD‐1 and DSM4166 both possess PhnD2, which showed a modest increase in abundance in both strains under Pi depletion (Supporting Information Fig. S4). The abundance of PhnD3 also increased in Pi‐deplete SBW25 and BIRD‐1 exoproteomes. PhnD4 is closely related to a SBP (SM_b21540) located upstream of genes (*phnWAY*) encoding an alternative pathway for the degradation of 2‐AEP in *Sinorhizobium meliloti* (Fig. 6A) (Borisova *et al*., 2011) and was only detected in Pi‐deplete BIRD‐1 cultures, albeit at a low abundance.

### Organic acid production

The Pi solubilization potential of the three Pseudomonads showed marked differences at both the genomic and exoproteomic level. Only DSM4166 induced secretion of two extracellular proteins annotated as pyroloquinoline quinone (PQQ)‐dependent alcohol dehydrogenases (QedH, PSTAA_2299; PedH, PSTAA_2293) in response to Pi stress (Fig. 3). Although BIRD‐1 does possess homologs of both of these proteins neither were detected in its exoproteome. All three strains expressed and secreted an exoprotein under Pi‐depletion that contained the Pfam07995 domain (glucose/sorbosone dehydrogenase) and this enzyme may represent a novel mechanism for extracellular organic acid production.

### Identification of novel Pi‐responsive exoproteins in *Pseudomonas* strains

In response to Pi‐depletion BIRD‐1 and SBW25 expressed a hypothetical outer membrane protein (PPUBIRD1_1354, PFLU4536) containing the Pfam16930 domain which is linked to porin structure (Figs. 3 and 4). In BIRD‐1 this was one of the most abundant proteins in the exoproteome of P‐stressed cells (Fig. 3). P‐stressed SBW25 cells secreted an uncharacterized lipoprotein containing a ParB‐like nuclease domain (Pfam08857), which may have a similar function to UshA (Rittmann *et al*., 2005) (Fig. 3). BIRD‐1 also secreted a putative extracellular phosphotriesterase/amidohydrolase (AmdDH3 II, PPUBIRD1_5046) that has the potential to cleave the C‐O‐P bond of certain pesticides (Sun *et al*., 2004). In DSM4166, three hypothetical exoproteins containing the domain of unknown function (DUF) 1329 increased in abundance in response to low Pi. However, these proteins did not increase in abundance in the exoproteomes of either of the other two *Pseudomonas* isolates. Finally, a number of putative extracellular proteases were enriched in the exoproteome of SBW25 in response to Pi depletion (Supporting Information Table S2).

### Analysis of the Pi‐responsive whole‐cell proteome in BIRD‐1

To gain a deeper understanding of the Pi‐responsive proteome, the whole‐cell proteome of BIRD‐1, which included both cytosolic and membrane protein fractions, was analysed. The majority of abundant proteins detected in the proteome of BIRD‐1 were housekeeping and central metabolism proteins (e.g. GyrB, DnaK, GroEL, RpoD, RpoB, FusA, IleS, GuaA, Tuf, SdhA, SdhB, AtpA, AtpB, RpsA, RpsC, SucC, SucA) whose abundance was not significantly affected by differing Pi regime (Supporting Information Table S4). A total of 267 proteins were significantly enriched [*t*‐test, *P* value ≤ 0.05, fold‐change (log2) ≥ 1.5] during Pi depletion and there was concordance between the two datasets. These enriched proteins included the transmembrane and ATP‐binding domains of the Pst system (PstC, PstA, PstB), a 2‐aminoethylphosphonate (2‐AEP)–specific phosphonatase (PhnX, PhnW) (Jiang *et al*., 1995; Baker *et al*., 1998; White and Metcalf, 2007), proteins involved in lipid remodelling (PlcP, DagK, OlsA, OlsB, Cfa, TauD) (Liu and Hulett, 1998; Antelmann *et al*., 2000; Zavaleta‐Pastor *et al*., 2010; Carini *et al*., 2015; Sebastian *et al*., 2016), a putative intracellular phosphatase (UxpA) and the twin‐arginine translocation (TAT) pathway (Putker *et al*., 2013) (Table 2). Proteins for both starch (MalQ, GlgE, GlgX, GlpA and GlpB) and polyhydroxyalkanoic acid (PhaA, PhaG, PhaC) biosynthesis (carbon storage) were also enriched during Pi stress (Supporting Information Table S4). The abundance of a cytoplasmic glucose‐6‐phosphate dehydrogenase (Zwf), as well as two distinct membrane‐bound (PPUBIRD1_4115, PPYBiRD1_2225) glucose dehydrogenases (Gcd, GcdII respectively), all of which are known to play a role in Pi solubilization through gluconic acid production (Miller *et al*., 2010; Roca *et al*., 2013), was also greater in Pi‐deplete cells. Finally, another HAD‐family phosphatase (encoded by PPUBIRD1_3492), similar to PhnX was only detected in the proteome of Pi‐deplete cells (Table 2).

**Table 2:** P‐responsive proteins in BIRD‐1 that are under the control of the PHO regulator, PhoBR.

Identified proteins		Accession number	Locus tag	Fold change – WT*	Fold change – mutant*
Proteins silenced in the *phoBR* mutant					
Hypothetical protein, conserved		ADR57845	PPUBIRD1_0136	4.12	ND
Taurine dioxygenase	*tauD*	ADR57960	PPUBIRD1_0256	2.31	ND
Phosphate‐specific methyl‐accepting chemotaxis protein	*ctpL*	ADR58302	PPUBIRD1_0612	4.95	ND
Dehydratase		ADR58320	PPUBIRD1_0630	2.32	ND
HlyD family type I secretion membrane fusion protein		ADR58534	PPUBIRD1_0849	3.42	ND
ABC transporter related protein		ADR58535	PPUBIRD1_0850	4.34	ND
lyso‐ornithine lipid acyltransferase	*olsA*	ADR58658	PPUBIRD1_0974	2.18	ND
ornithine‐acyl[acyl carrier protein] N‐acyltransferase	*olsB*	ADR58659	PPUBIRD1_0975	4.14	ND
L‐serine dehydratase		ADR58719	PPUBIRD1_1037	2.05	ND
Arginine/ornithine antiporter	*arcD*	ADR58734	PPUBIRD1_1052	3.04	ND
General secretion pathway protein K	*tatA*	ADR58773	PPUBIRD1_1091	3.34	ND
Alkaline phosphatase	*phoX*	ADR58775	PPUBIRD1_1093	5.14	ND
2',3'‐cyclic‐nucleotide 2'‐phosphodiesterase	*uxpA*	ADR58776	PPUBIRD1_1094	3.24	ND
General secretion pathway protein G	*tatG*	ADR58781	PPUBIRD1_1099	4.60	ND
Hypothetical protein, conserved		ADR59032	PPUBIRD1_1354	5.10	ND
Metallophosphoesterase	*plcP*	ADR59065	PPUBIRD1_1390	4.59	ND
Gluconate 2‐dehydrogenase acceptor subunit		ADR59804	PPUBIRD1_2165	3.15	ND
2Fe‐2S iron‐sulfur cluster binding domain		ADR59806	PPUBIRD1_2167	4.47	ND
Probable quinate dehydrogenase	*gcdII*	ADR59861	PPUBIRD1_2225	3.26	ND
UDP‐glucose 4‐epimerase		ADR60229	PPUBIRD1_2604	2.14	ND
Response regulator		ADR60354	PPUBIRD1_2733	3.33	ND
UDP‐glucose 6‐dehydrogenase	*tuaD*	ADR60422	PPUBIRD1_2809	5.63	ND
Putative cyclopropane fatty acid synthase A	*cfa2*	ADR60552	PPUBIRD1_2940	2.93	ND
Hypothetical protein, conserved		ADR60627	PPUBIRD1_3016	4.96	ND
Phosphate ABC transporter, ATP‐binding domain	*pstB1*	ADR60628	PPUBIRD1_3017	6.70	ND
Phosphate ABC transporter, transmembrane domain	*pstA1*	ADR60629	PPUBIRD1_3018	4.52	ND
Phosphate ABC transporter transmembrane domain	*pstC1*	ADR60630	PPUBIRD1_3019	3.91	ND
Phosphate ABC transporter, periplasmic binding domain	*pstS1*	ADR60631	PPUBIRD1_3020	5.08	ND
2‐aminoethylphosphonate–pyruvate transaminase	*phnW*	ADR61037	PPUBIRD1_3442	3.87	ND
Phosphonoacetaldehyde hydrolase	*phnX*	ADR61038	PPUBIRD1_3443	4.76	ND
Hypothetical protein, conserved (HAD‐like domain)		ADR61085	PPUBIRD1_3492	3.081	ND
Cation/acetate symporter actP	*actP*	ADR61460	PPUBIRD1_3874	2.74	ND
GntR family transcriptional regulator		ADR61476	PPUBIRD1_3890	3.57	ND
ABC transporter ATP‐binding protein		ADR61481	PPUBIRD1_3895	4.72	ND
Metallopeptidase, zinc binding protein		ADR61927	PPUBIRD1_4353	2.26	ND
Fe3+ ABC transporter, periplasmic binding domain		ADR62476	PPUBIRD1_4925	2.04	ND
Fe3+ ABC transporter, ATP‐binding domain		ADR62478	PPUBIRD1_4927	2.85	ND
Arylesterase, putative		ADR62594	PPUBIRD1_5047	3.47	ND
Winged helix family regulator	*phoB*	ADR62659	PPUBIRD1_5112	4.38	ND
Phosphate regulon sensor protein	*phoR*	ADR62660	PPUBIRD1_5113	4.64	ND
Phosphate ABC transporter, ATP‐binding domain	*pstB2*	ADR62665	PPUBIRD1_5118	5.16	ND
Phosphate ABC transporter, transmembrane domain	*pstA2*	ADR62666	PPUBIRD1_5119	4.93	ND
Phosphate ABC transporter, transmembrane domain	*pstC2*	ADR62667	PPUBIRD1_5120	6.46	ND
Phosphate ABC transporter, periplasmic binding domain	*pstS2*	ADR62668	PPUBIRD1_5121	3.55	ND
Proteins that were down‐regulated compared with the WT					
Taurine ABC transport, periplasmic binding domain	*tauA*	ADR57963	PPUBIRD1_0259	3.10	−1.14
Quinoprotein glucose dehydrogenase A	*gcd*	ADR61697	PPUBIRD1_4115	2.03	0.86
Methyl‐accepting chemotaxis sensory transducer		ADR61928	PPUBIRD1_4354	1.99	−0.38
Phosphate transport regulator	*phoU*	ADR62664	PPUBIRD1_5117	2.07	−0.32
Proteins whose expression was not affected by PhoBR					
Cyclopropane‐fatty‐acyl‐phospholipid synthase	*cfa1*	ADR57745	PPUBIRD1_0035	2.71	4.55
Phosphonate ABC transporter, periplasmic binding domain	*phnD2*	ADR58557	PPUBIRD1_0873	0.62	1.16
ABC‐type Fe3+ transport system periplasmic binding domain	*phnD3*	ADR61477	PPUBIRD1_3891	2.69	2.31
Exopolyphosphatase	*ppX*	ADR62560	PPUBIRD1_5012	0.44	0.66
Polyphosphate kinase	*ppK*	ADR62561	PPUBIRD1_5013	1.34	0.89
Amidohydrolase 3	*amdhd3II*	ADR62593	PPUBIRD1_5046	4.45	3.97
PhoH family protein	*phoH*	ADR61846	PPUBIRD1_4270	1.38	2.78

ND, not detected; WT, wild type.

A number of P‐responsive proteins of interest that are not regulated by PhoBR are also listed. The abundance of these proteins within the Proteome of BIRD‐1 is shown in Table S4. The accession number shown refers to the Uniprot database. * represents Log2 transformation of fold change values that are the mean of triplicate cultures. All proteins displayed in the Table with a Log2 value ≥ 1.5, were also statistically significantly enriched under Pi depletion (*t*‐test, *P* value ≤ 0.05).

### Identification of PHO‐regulated Pi‐responsive proteins in BIRD‐1

To assess how the Pi‐responsive proteome and exoproteome in BIRD‐1 is regulated we disrupted the genes encoding the master regulator of the PHO regulon, *phoBR*. Compared with the wild type, the *phoBR* mutant showed a substantial reduction in final growth yield when grown under Pi deplete, but not Pi replete conditions (Supporting Information Fig. S5). As with the wild type strain, we performed exoproteome and whole‐cell proteome analysis for the *phoBR* strain. In the *phoBR* mutant, three categories of Pi responsive proteins were determined: (1) proteins that were absent (below detection level) in the mutant and observed in the wild type (2) proteins that no longer increased in abundance under Pi stress and (3) proteins whose abundance in both Pi‐replete and Pi‐deplete growth conditions was similar to the wild type. In the whole‐cell proteome, PstSCAB1&2, PhoX, UxpA, PhoBR, PhoU, TatADG, PhnXW, PlcP, OlsAB, TuaD were all absent during Pi‐deplete growth of the mutant (Table 2). With respect to Pi solubilization, GcdII was also absent, whereas Gcd was not enriched in Pi‐deplete cells, unlike the wild type. However, the abundance of Zwf was unaffected by mutation of *phoBR*. The putative HAD‐like phosphatase (PPUBIRD‐1_3492), as well as the hypothetical outer membrane protein PPBUBIRD1_1354, were also absent in the mutant, suggesting that these two proteins are novel members of the pho regulon (Table 2). Interestingly, AmdHd3II was still enriched during Pi‐stress suggesting that it is not regulated by *phoBR*. The abundance of several other proteins was also not affected by mutation of *phoBR* (Table 2 and Supporting Information Table S5) indicating that a PHO‐independent response to Pi stress occurs in BIRD‐1. For example, all the carbon storage proteins and proteins linked with biofilm formation (encoded by PPUBIRD1_2591‐2608) still showed a PHO‐independent response to Pi stress.

In the exoproteome of the BIRD‐1 *phoBR* mutant, PstS1, PhoX, the hypothetical exoprotein/porin (PPUBIRD1_1354), PhnD4 were all absent from either growth condition. Meanwhile, the abundance of PstS2 PhnD2, PhnD3, GSDH, AmdHd3, AmdHdII and the hypothetical exoprotein (PPUBIRD1_3958) were all reduced in Pi deplete cultures compared with the wild type, but were still detected (Figs. 3 and 4).

## Discussion

Characterizing the exoproteomes, and thus the functional entities associated with environmental interactions (Armengaud *et al*., 2012), of various *Pseudomonas* strains allowed us to deepen our understanding of Pi‐regulated protein expression in this genus (Figs. 3 and 4). While, genomic comparisons, based on known proteins in the literature, allowed us to access the heterogeneity in their P‐mobilizing and P‐scavenging ‘potential’, proteomic analyses revealed both differences in their global regulatory networks and also helped to identify novel Pi‐responsive proteins, which may be of further biotechnological interest. For example, a novel Pi‐responsive extracellular nuclease in SBW25 was discovered that was not identified through our comparative genomic analysis. Importantly, the genes (PPUBIRD1_5077, PPUBIRD1_0727, PPUBIRD1_2395, PPUBIRD1_0951, PPUBIRD1_0932) identified in BIRD‐1, based solely on *in silico* annotation (Roca *et al*., 2013), were not members of the PHO regulon, highlighting the need for auxiliary studies to confirm genomic annotation. Furthermore, the strong secretion of exoproteins, such as PstS and PhoX, may serve as markers for characterizing complex communities in soil/rhizosphere to enable identification of the key microbial taxa involved in P recycling.

Soil organic P exists in many forms and frequently accounts for 30%–65% of total P in soils (Harrison, 1987) and its mineralization to Pi can have a great impact on total P bioavailability (Turner *et al*., 2002; Shen *et al*., 2011). From the genomic comparison of each strain, it appears that DSM4166 has the greatest ability to degrade organic P compounds as it contains two distinct PhoX homologs as well as PhoD, UshA and GlpQ (Larson *et al*., 1983; Antelmann *et al*., 2000; Rittmann *et al*., 2005; Monds *et al*., 2006; Pinchuk *et al*., 2008; Putker *et al*., 2013). We also identified an UshA‐like homolog and a GlpQ‐like homolog (PFLU4789) in the genomes of BIRD‐1 and SBW25, respectively, but in contrast to DSM4166, neither or these homologs were secreted in response to Pi depletion. SBW25 also heavily secreted Psp, a phosphate‐binding protein (Scott and Wu, 2005), which is closely related to the low molecular weight phosphatases, LapA and LapB, in *P. aeruginosa* (Tan and Worobec, 1993; Ball *et al*., 2002). Psp may, therefore, be the exoenzyme responsible for the unnaccounted APase activity detected in *P. fluorescens* Pf0‐1 (Monds *et al*., 2006). Interestingly, it was SBW25 that elicited the strongest APase activity towards *p*NPP (phosphomonoesterase activity). To date, little is known about the natural substrate range of these promiscuous enzymes in soil and it is likely that differences occur between the different PhoX homologs. In support of this hypothesis, SBW25 PhoX is phylogenetically distinct (Supporting Information Fig. S6) from either BIRD‐1 or DSM4166 homologs.

Although P solubilization through organic acid production has been well studied in *Pseudomonas* (Rodrı'guez and Fraga, 1999; Miller *et al*., 2010) several putatively new P solubilizing proteins likely involved in this process were still identified at the genomic level, e.g. QedH, PedH, and an alternative PHO‐regulated Gcd, GcdII (Fig. 2; Table 2). However, a similar discordance between genomic prediction and proteomic abundance was observed for Pi‐solubilizing enzymes. For example, QedH and PedH were detected in Pi‐depleted DSM4166 cultures, but not in BIRD‐1. GSDH, another previously uncharacterized protein with respect to the PHO regulon, and not identified in our genomic assessment, was secreted in all three strains in response to Pi‐depletion and likely has a role in Pi‐solubilization though organic acid release. The lower abundance of Pi‐solubilizing proteins may have resulted from an absence of glucose in the growth medium the precursor substrate for these Pi‐solubilizing enzymes.

Although the three *Pseudomonas* strains can grow on phytate as a source of P (Lim *et al*., 2007; Roca *et al*., 2013), there was no evidence that either the known phytase of DSM4166 and SBW25, or the predicted phytase, responsible for the growth of BIRD‐1 on this substrate (Roca *et al*., 2013) are regulated by PhoBR. Considering that phytate is usually ubiquitous in soils (Stutter *et al*., 2012), Pseudomonads have likely adapted to express their respective phytases solely in response to the presence of this compound and may explain why these Pi‐mobilizing enzymes are unexpectedly not part of the *Pseudomonas* PHO regulon.

The existence of two distinct Pst systems in both BIRD‐1 and SBW25 is similar to that of *Synechocystis*, *Vibrio cholerae* and the Archaeon, *Halobacterium salinarium* R1 (Furtwängler *et al*., 2010; Pitt *et al*., 2010; Mudrak and Tamayo, 2012). As Pst2 is present in all *Pseudomonas* strains, while Pst1 appears in only a few, we hypotheize that Pst2 is essential for efficient uptake of Pi in *Pseudomonas*. However, Pst1 must clearly have a role in Pi uptake as we detected PstS1 as well as PstS2 in both BIRD‐1 and SBW25 exoproteomes. Furthermore, although disruption of PstS1 in SBW25 did not affect growth on low Pi in isolation, it did confer a fitness reduction in the presence of the wild type (Zhang *et al*., 2007). In *Synechocystis, Vibrio* and *H. salinarium*, Pst1 and Pst2 either have different kinetic parameters for the uptake of Pi (Furtwängler *et al*., 2010; Pitt *et al*., 2010) or are expressed during different growth phases (planktonic v biofilm) (Pratt *et al*., 2010; Mudrak and Tamayo, 2012). The data presented in this study favours the hypothesis that they have different kinetic parameters as both were expressed in BIRD‐1 and SBW25 during planktonic growth.

Only BIRD‐1 and SBW25 have the genetic potential to catabolize phosphonates (Table 1), and in BIRD‐1, phosphonatase (PhnWX) was PHO‐regulated. Based on our data, we cannot rule out the possibility that DSM4166 can also grow on phosphonates as a source of P for two reasons: (i) Although phosphonate degradation has been well documented in recent years (Jiang *et al*., 1995; White and Metcalf, 2007; Villarreal‐Chiu *et al*., 2012; McGrath *et al*., 2013), bacteria capable of growing on phosphonates that do not possess any of the characterized genes/proteins have been isolated, demonstrating alternative pathways for phosphonate degradation must exist in nature (Fox and Mendz, 2006); (ii) DSM4166 possesses and expressed a number of putative phosphonate transporters (Table 1 and Fig. 6). Characterizing these putative transporters will surely enhance our knowledge regarding phosphonate degradation in *Pseudomonas* strains and may provide new molecular markers for investigating the *in situ* cycling of these compounds (Mauchline *et al*., 2006; Christie‐Oleza and Armengaud, 2010; Lidbury *et al*., 2014).

Bacteria that can remodel their lipid membranes in order to reduce their ratio of P‐containing:non P‐containing lipids (Zavaleta‐Pastor *et al*., 2010; Carini *et al*., 2015; Sebastian *et al*., 2016) are desirable to use as PGPR as their requirement for P is reduced. We found genes encoding for the key proteins required for lipid remodelling (PlcP, DagK, OlsA and OlsB) in the genomes of all *Pseudomonas* strains scrutinised (Gao *et al*., 2004; Zavaleta‐Pastor *et al*., 2010). Furthermore, in BIRD‐1 these proteins were expressed in a PHO‐dependent manner. Interestingly, in BIRD‐1 UDP‐glucose 6‐phosphate dehydrogenase (TuaD) was also PHO‐regulated. In *B. subtilis*, TuaD is encoded by the *tua* operon that is involved in the production of teichuronic acid lipids during Pi‐depletion (Liu and Hulett, 1998; Antelmann *et al*., 2000). The rest of the genes required for teichuronic acid were absent, therefore, making the role of TuaD somewhat unclear in BIRD‐1. As all of these proteins were silenced in the *phoBR* mutant, it is likely that remodelling the lipid membrane accounts for the gross difference observed in growth between this strain and the wild type when grown under Pi‐deplete conditions (Fig. 7).

The observed heterogeneity in the Pi responsive portion of the proteome of the three *Pseudomonas* strains in this study highlights how the utilization of different PGPR can have potentially different effects in the rhizosphere. For example, based on our genomic and proteomics data, differences in the ability of the strains to degrade phospholipids, nucleic acids and organopesticides (Singh and Walker, 2006; Bigley and Raushel, 2013), as well as a likely difference in their broad organic P substrate range may have marked effects on their ability to mobilize P for a plant host under certain environmental conditions. For example, the addition of a strain comparable to DSM4166, expressing UshA and GlpQ, may increase the acquisition of P in plants when using manure as the nutrient source, which is rich in nucleic acids and phospholipids (Turner and Leytem, 2004; Shen *et al*., 2011). Furthermore, DSM4166 is a known nitrogen fixer and in certain soils (nitrogen‐limited) employing this strain over that of either BIRD‐1 or SBW25 may provide more efficient plant‐growth promotion. Likewise, in soils contaminated with phosphorus‐containing organopesticides, it may be more suitable to deploy a strain similar to SBW25, which contains the promiscuous C‐P lyase, capable of degrading these compounds, or BIRD‐1 which possesses a Pi‐responsive putative phosophotriesterase.

## Conclusions

Observing the global exoproteomic response of just three *Pseudomonas* species revealed new insights into the P scavenging capabilities of this genus and has provided a number of markers (Muth *et al*., 2015) that can be utilized to investigate P‐mobilization directly in the rhizosphere. Given the enormous task of identifying proteins *in situ* from complex communities, the data presented in this paper will serve as a platform to investigate the key enzymes and microbial taxa involved in P‐mobilization at the level of functional entities (proteins) *in situ*. Meta‐exoproteomics has already identified differences between genomic and proteomic assessments of soil chintase‐degrading communites (Johnson‐Rollings *et al*., 2014) and should also shed light on the ‘black box’ concerning P‐mobilization in the rhizosphere.

## Experimental procedures

### Growth and maintenance of bacterial strains

All three *Pseudomonas* strains were maintained on Luria Bertani (LB) agar (1.5% w/v) medium at 30°C. To investigate the effect of Pi‐depletion on the three strains, each was grown (*n* = 3) in an adapted Minimal A medium comprising: Na‐Succinate 5.4 g l^−1^, NaCl 200 mg l^−1^, NH_4_Cl 450 mg l^−1^, CaCl_2_ 200 mg l^−1^, KCL mg l^−1^ MgCl_2_ 450 mg l^−1^, FeCl_2_ 10 mg l^−1^, MnCl_2_ 10 mg l^−1^, 10 mM 4‐(2‐hydroxyethyl)−1‐piperazineethanesulfonic acid (HEPES) pH 7.2, with KH_2_PO_4_ added to a final concentration of either 50 μM or 1.4 mM. Each strain was pre‐cultured in minimal A medium containing 400 μM Pi to ensure cells had adequate Pi while minimizing the potential for carry over of residual Pi into triplicate experimental cultures.

### Quantification of alkaline phosphatase activity

A 0.5 ml culture (*n* = 3) was incubated with 20 μl *para*‐nitrophenyl phosphate (*p*NPP) (final conc. 4mM) and incubated at room temperature for 1 h or when colour development started to occur. The reaction was stopped using 25 μl NaOH (2 mM) and incubated for 10 min. Cell debris and precipitants were removed via centrifugation (2 min, 8,000 × *g*) prior to spectrophotometry (optical density 405 nm). A standard curve for *para*‐nitrophenol was generated using a range of known concentrations (0, 4, 8, 25, 50, 75, 100 mg ml^−1^).

### Preparation of exoproteomes, trypsin in‐gel proteolysis, nano‐LC‐MS/MS analysis and peptide identification through MS/MS database searching

Exoproteomes were analysed using modified methods of Christie‐Oleza and Armengaud (2010). The recorded MS/MS spectra were searched against the protein sequence database (*P. putida* BIRD‐1, NC_017530.1; *P. fluorescens* SBW25, NC_012660.1; *P. stutzeri* DSM4166, NC_017532.1). Full details of the protocol and parameters used for peptide identification can be found in the supplementary materials and methods.

### Quantification of detected proteins

The Normalized Spectral Abundance Factor (NSAF) values were calculated using SCAFFOLD v4.0 according to software defaults. For the exoproteomes, no further normalization was performed. For whole‐cell proteomics, 25 μg of protein was loaded onto SDS‐PAGE gels prior to identification. No further normalization was performed. However, we examined the abundance of several housekeeping proteins and central metabolic enzymes and did not observe substantial change in their abundance. For determining the proportion of proteins within the exoproteome, replicate cultures (*n* = 3) were averaged. The proteomics data has been deposited in the Proteomics Identification (PRIDE) database (Martens *et al.*, 2005) with the following accession numbers: PXD004065, PXD004064, PXD003830, PXD003829, PXD003828, PXD003827, PXD003826.

### Bioinformatics analysis of detected proteins and comparative genomics

The majority of analyses were performed using the Integrated Microbial Genomes Database at the Joint Genome Institute (IMG/JGI) server (http://img.jgi.doe.gov/). Please refer to supplementary information for a detailed summary of the bioinformatics approaches used. IMG/JGI was also used for comparative genomic analyses. BLASTP (expected value, e‐30, minimum identity = 20%) searches were performed using the proteins detected in the exoproteomes/proteome. In some cases other proteins identified from the literature known to be involved in Pi scavenging/recycling were used as queries for BLASTP analysis. For the Pi‐binding proteins (PBP), a function search using the IMG/JGI database was performed using the Pfam domain, 12849 as the query.

### Genetic manipulation of *P. putida* BIRD‐1

To construct a *phoBR* mutant of *P. putida* BIRD‐1, the method outlined by Lidbury *et al*. (2014) was adapted. Please refer to the supplementary information for a detailed procedure.

## Supporting information

Additional Supporting Information may be found in the online version of this article at the publisher's web‐site:

**Fig. S1**. Proportion of the extracellular and intracellular proteins detected in the four protein fractions extracted during this study. Only the top‐60 most abundant proteins were included in the analyses. The values displayed are taken from Pi‐deplete and Pi‐replete cultures. Results presented are the mean of triplicate cultures.
**Fig. S2**. The abundance of proteins in both the high Pi and low Pi exoproteomes of the three *Pseudomonas* strains. **(A)** *Pseudomonas fluorescens* SBW25, **(B)** *Pseudomonas putida* BIRD‐1, **(C)** *Pseudomonas stutzeri* DSM4166. Results are the mean of triplicate cultures and error bars denote standard deviation.
**Fig. S3.** Conservation of the key residues (highlighted in red) involved in phosphate binding among the periplasmic binding proteins containing the domain, Pfam12849‐ PBP. Locus tags are used as the identifier. Abbreviations: VP, *Vibrio parahaemolyticus*; VC/VCA, *V. cholerae*; VAA, *V. anguillarum*; *V. harveyi* MYO, *Synechocystis* sp. PCC6803; PFLU, *P. fluorescens*; PA, *P. aeruginosa*; PPUBIRD1, *P. putida*; PSTAA, *P. stutzeri*; Psyr, *P. syringae*; EcDH1, *E. coli*; *P. Antarctica*; Smc; *Ensifer meliloti*.
**Fig. S4.** Semi‐quantitative abundance analysis of the putative phosphonate substrate binding proteins detected in the exoproteomes of the three *Pseudomonas* strains. and *the phoBR* mutant. Results presented are the mean of triplicate cultures. Error bars denote standard deviation.
**Fig. S5** Growth of the *phoBR* mutant strain of *P. putida* BIRD‐1. **A.** A comparison of the *phoBR* mutant grown under Pi‐replete (Black circles) and Pi‐deplete (Grey circles) conditions. Concentrations of Pi were the same as those used for the wild type. Black arrows indicated the times of sampling for proteomics and exoproteomics. The striped arrow indicates the addition of Pi (50 μM) to help generate enough biomass for sampling. **B.** Growth yields of either the wild type or *phoBR* mutant sampled after 48 hours grown on Pi‐replete or Pi‐deplete growth media. Results presented are the mean of triplicate cultures. Error bars denote standard deviation.
**Fig. S6**. Evolutionary relationships of PhoX‐like homologs. The evolutionary history was inferred using the Neighbor‐Joining method [1]. The optimal tree with the sum of branch length = 3.66263884 is shown. The tree is drawn to scale, with branch lengths in the same units as those of the evolutionary distances used to infer the phylogenetic tree. The evolutionary distances were computed using the p‐distance method [2] and are in the units of the number of amino acid differences per site. The analysis involved 27 amino acid sequences. All ambiguous positions were removed for each sequence pair. There were a total of 842 positions in the final dataset. Evolutionary analyses were conducted in MEGA6 [3].

**Table S1**. A rank‐abundance profile of the identified proteins in the exoproteome of *Pseudomonas putida* BIRD‐1.
**Table S2**. A rank‐abundance profile of the identified proteins in the exoproteome of *Pseudomonas fluorescens* SBW25.
**Table S3**. A rank‐abundance profile of the identified proteins in the exoproteome of *Pseudomonas stutzeri* DSM4166.
**Table S4**. A rank‐abundance profile of the identified proteins in the proteome of *P. putida* BIRD‐1.
**Table S5**. A rank‐abundance profile of the identified proteins in the cellular proteome of the *P. putida* BIRD‐1 *phoBR* mutant.
**Table S6.** A rank‐abundance profile of the identified proteins in the exoproteome of the *P. putida* BIRD‐1 *phoBR* mutant.
