# Supplementary material for: Comparative genomic, proteomic and exoproteomic analyses of three Pseudomonas strains reveals novel insights into the phosphorus scavenging capabilities of soil bacteria
Source: Environ Microbiol. 2016 Jul 7;18(10):3535–49. doi: 10.1111/1462-2920.13390 (PMC5082522; doi:10.1111/1462-2920.13390)
Supplement: Supplementary file 1 — Fig. S1. Proportion of the extracellular and intracellular proteins detected in the four protein fractions extracted during this study. Only the top‐60 most abundant proteins were included in the analyses. The values displayed are taken from Pi‐deplete and Pi‐replete cultures. Results presented are the mean of triplicate cultures. Fig. S2. The abundance of proteins in both the high Pi and low Pi exoproteomes of the three Pseudomonas strains. (A) Pseudomonas fluorescens SBW25, (B) Pseudomonas putida BIRD‐1, (C) Pseudomonas stutzeri DSM4166. Results are the mean of triplicate cultures and error bars denote standard deviation. Fig. S3. Conservation of the key residues (highlighted in red) involved in phosphate binding among the periplasmic binding proteins containing the domain, Pfam12849‐ PBP. Locus tags are used as the identifier. Abbreviations: VP, Vibrio parahaemolyticus; VC/VCA, V. cholerae; VAA, V. anguillarum; V. harveyi MYO, Synechocystis sp. PCC6803; PFLU, P. fluorescens; PA, P. aeruginosa; PPUBIRD1, P. putida; PSTAA, P. stutzeri; Psyr, P. syringae; EcDH1, E. coli; P. Antarctica; Smc; Ensifer meliloti. Fig. S4. Semi‐quantitative abundance analysis of the putative phosphonate substrate binding proteins detected in the exoproteomes of the three Pseudomonas strains. and the phoBR mutant. Results presented are the mean of triplicate cultures. Error bars denote standard deviation. Fig. S5 Growth of the phoBR mutant strain of P. putida BIRD‐1. A. A comparison of the phoBR mutant grown under Pi‐replete (Black circles) and Pi‐deplete (Grey circles) conditions. Concentrations of Pi were the same as those used for the wild type. Black arrows indicated the times of sampling for proteomics and exoproteomics. The striped arrow indicates the addition of Pi (50 μM) to help generate enough biomass for sampling. B. Growth yields of either the wild type or phoBR mutant sampled after 48 hours grown on Pi‐replete or Pi‐deplete growth media. Results presented are the mean of tripl [file EMI-18-3535-s001.docx]

**Supplementary information**

**Materials and Methods**

**Preparation of exoproteomes, trypsin in-gel proteolysis and nano-LC-MS/MS analysis**

The supernatants of bacterial cultures were harvested during mid exponential growth (OD_600_ 0.5 - 0.8). Cells were removed via centrifugation (3,200 x *g*, 10 min) and filtration through a 0.22 μm membrane (Fisherbrand, syringe filter PVDF 33mm). 40 ml of supernatant was pre-filtered through a 0.45 μm membrane (Fisherbrand, syringe filter PVDF 33mm). Proteins in the remaining milieu were concentrated and purified by precipitation with trichloroacetic acid (75% w/v) and separated using SDS-polyacrylamide gel electrophoresis (SDS-PAGE) following the methods of (Christie-Oleza and Armengaud, 2010). A modified final volume of 60 μl RunBlue lithium dodecyl sulphate (LDS) loading buffer (Expedeon) was used to dissolve the protein pellet. 25 μl of precipitated proteins were loaded onto a 4-20 % SDS Precast gel (Expedeon). SDS-PAGE was performed by using 20X Teo-Tricine-SDS running buffer (Expedeon) for at 180 V for 45 min. Gels were stained using Instant Blue (Expedeon). For BIRD-1 wild type and *phoBR* mutant a short run was performed and a single polyacrylamide gel band containing the entire exoproteome was cut and processed for in-gel proteolysis with trypsin (Roche) as previously described (Christie-Oleza and Armengaud, 2010). For SBW25 and DSM4166, the entire gel lane was separated into 5 distinct slices prior to processing.

**Peptide identification through MS/MS database searching**

The recorded MS/MS spectra were searched against the protein sequence database (*P. putida* BIRD-1, NC_017530.1; *P. fluorescens* SBW25, NC_012660.1; *P. stutzeri* DSM4166, NC_017532.1). The search was carried out with MASCOT 2.2.04 software (Matrix Science). Parameters were established as follows: tryptic peptides with a maximum of 1 missed cleavage during proteolytic digestion, a mass tolerance of 5 ppm on the parent ion and 0.5 Da on the MS/MS, fixed modification for carboxyamidomethylated Cysteine and variable modification for oxidized Methionine. MASCOT results were combined and parsed using Scaffold (Proteome Software). A protein identification was considered valid when at least two different peptides were detected in the same experiment. The false-positive rate for protein identification was estimated using a reverse decoy database as below 0.1% with these parameters by peptide prophet as part of Scaffold.

**Bioinformatic analysis of detected proteins**

Proteins and their corresponding open reading frames (ORFs) were analysed using the Intergrated Microbial Genomes Database at the Joint Genome Institute (IMG/JGI) (http://img.jgi.doe.gov/). Proteins not containing a signal peptide leader sequence where analysed using the online server SecretomeP 2.0 (<http://www.cbs.dtu.dk/services/SecretomeP/>). Where applicable, proteins of interest were used as queries in homology searches performed using HMMR (<http://www.ebi.ac.uk/Tools/hmmer/>). BLAST searches were performed using the IMG/JGI database against selected genomes using a cut off >20% identity (amino acid) and an expected value E < -20. To identify phosphate binding protein homologs, a function search was also performed in the IMG/JGI server using Pfam01248 as the query. Phylogenetic analysis was conducted using the freeware, MEGA 6.1 (Tamura et al., 2011).

**Genetic manipulation of *P. putida* BIRD-1**

To construct a *phoBR* mutant of *P. putida* BIRD-1*,* we adapted the methods outlined by Lidbury et al., (2014). Initially, two regions of genomic DNA were PCR amplified, one toward the 5′ end of *phoB* (Primers: phoBRAF, 5- attcgagctcggtacccgggCGGCCTTCTTGTCCTTGATG-3; phoBRAR, 5- tagagtcgacTCCTGCCTACCATGCTCAA-3) and the other toward the 3′ end of *phoR* Primers: phoBRBF – 5- cggccaattcCATCAAGAACGACGCCCAG-3; phoBRBR, 5- cctgcaggtcgactctagagAATGTAGCGGCTGGGGCT-3), along with the gentamicin resistance cassette (Primers: phogentF, 5- gtaggcaggaGTCGACTCTAGAGGATCCCCG, phogentR, 5’ cctgcaggtcgactctagagAATGTAGCGGCTGGGGCT-3) from p34S-Gm (Dennis and Zylstra, 1998). The suicide vector pK18mob*sacB* (Schäfer et al., 1994) was linearised using the restriction enzyme *BamH*I and all four fragments of DNA were ligated together using Gibson Cloning (New England Biolabs, Hitchin, UK) and the manufacturer’s guidelines. The resulting plasmid was transformed into *Escherichia coli* S17.1 via electroporation and mobilized into *P. putida* BIRD-1 via conjugation (3 h @ 30^O^C) on a 0.22 µm pore-size, 47 mm sterile filter (Millipore, UK), using LB as the medium. Transconjugants were selected on LB containing gentamicin (50 g ml^-1^) and using chloramphenicol (10 g ml^-1^) as the counter selection against *E. coli*. A single crossover transconjugants was grown overnight in LB and plated onto LB containing gentamicin and 10% sucrose to select for double crossover mutants. Homologous recombination was confirmed by PCR and DNA sequencing.

**Figure S1**. Proportion of the extracellular and intracellular proteins detected in the four protein fractions extracted during this study. Only the top-60 most abundant proteins were included in the analyses. The values displayed are taken from Pi-deplete and Pi-replete cultures. Results presented are the mean of triplicate cultures.


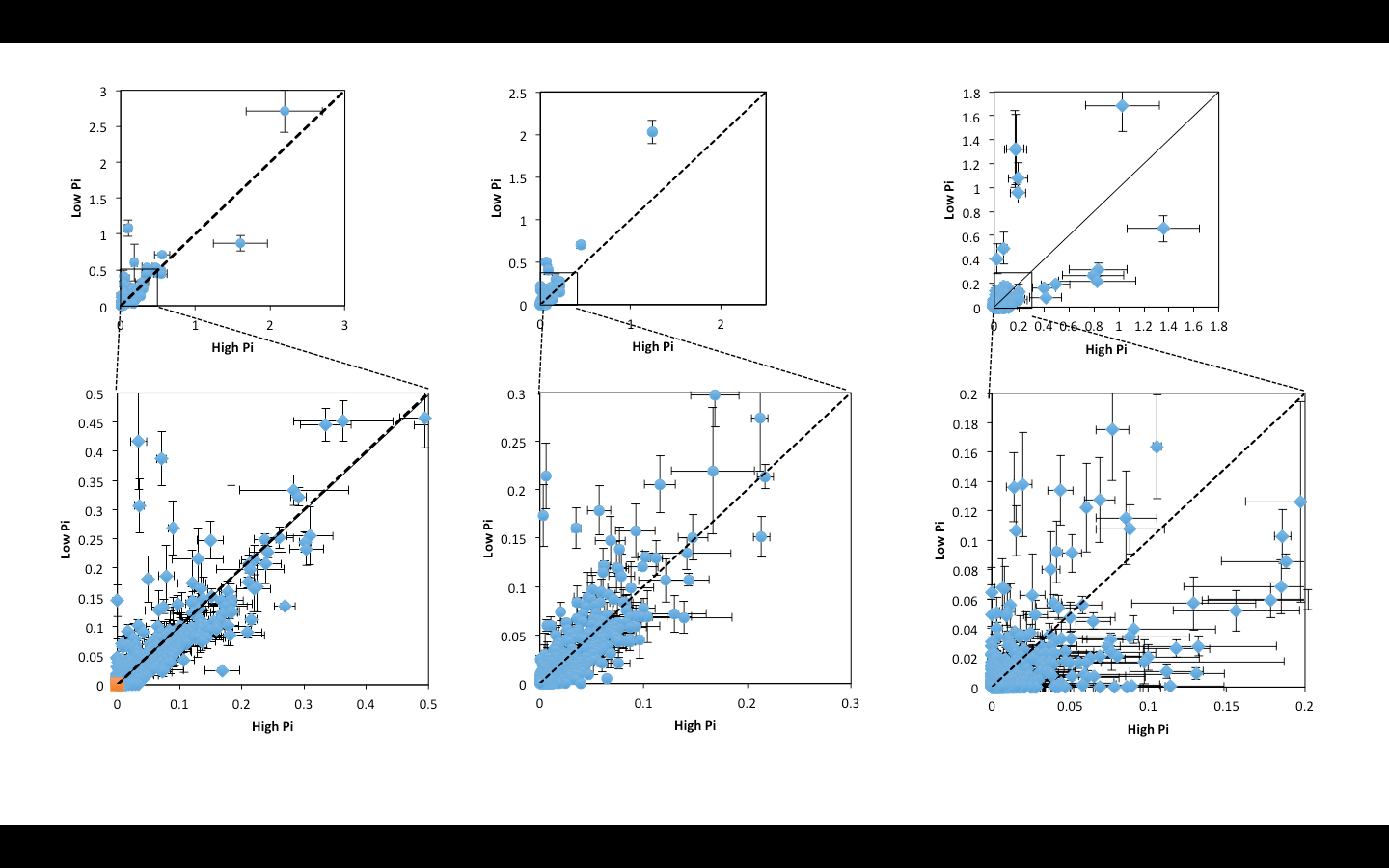
A) B) C)

**Figure S2**. The abundance of proteins in both the high Pi and low Pi exoproteomes of the three *Pseudomonas* strains. **(A)** *Pseudomonas fluorescens* SBW25, **(B)** *Pseudomonas putida* BIRD-1, **(C)** *Pseudomonas stutzeri* DSM4166. Results are the mean of triplicate cultures and error bars denote standard deviation.


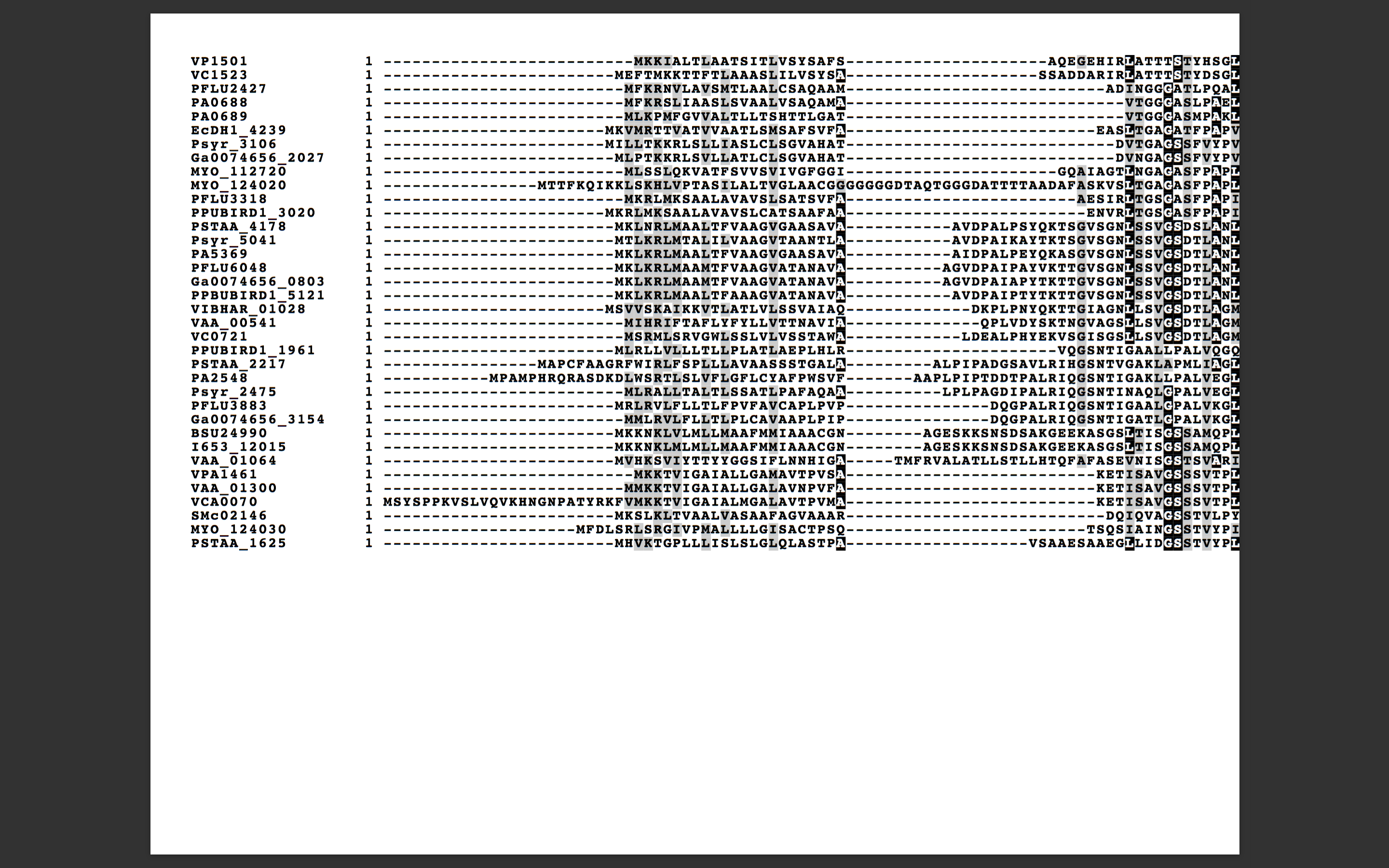

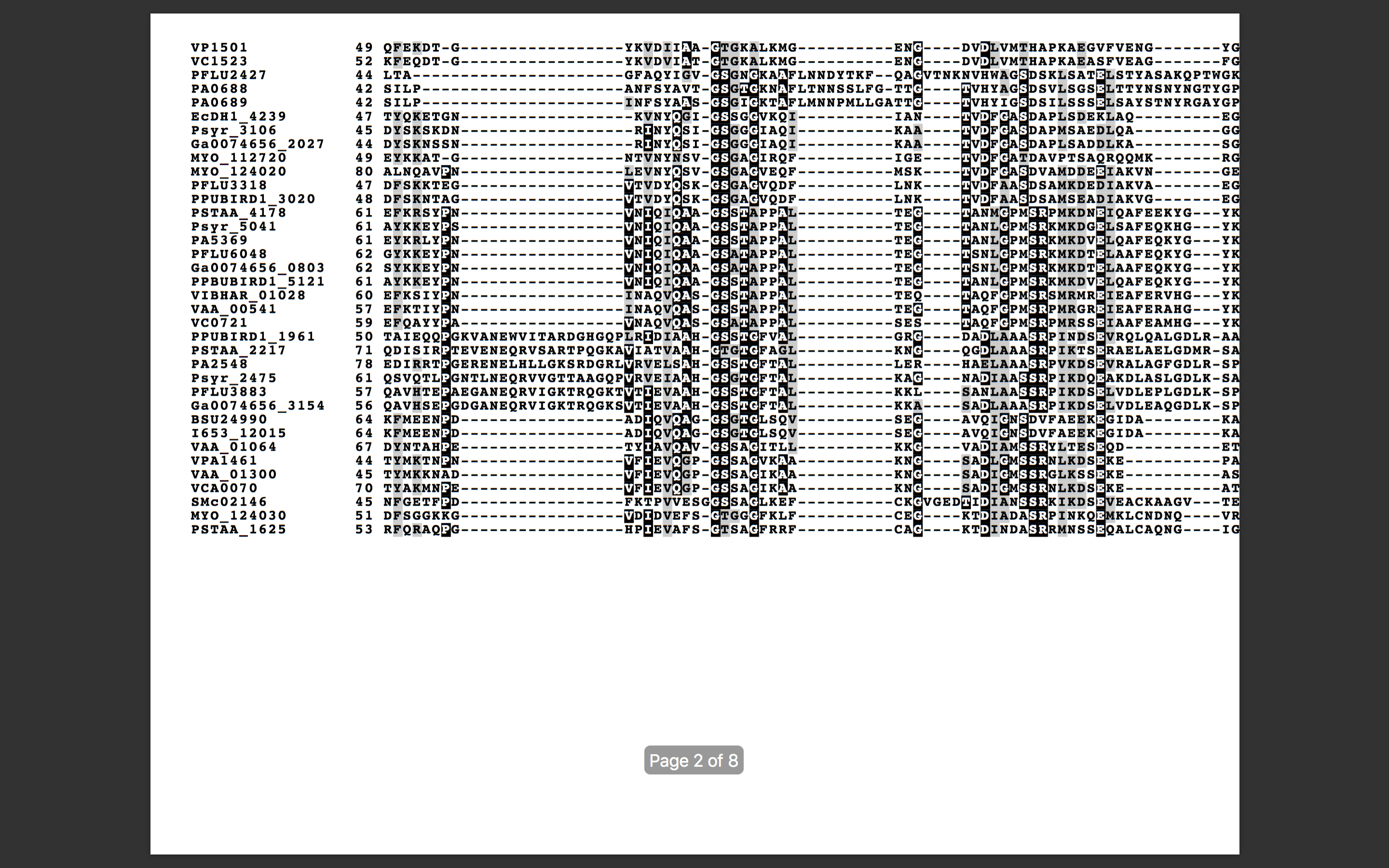

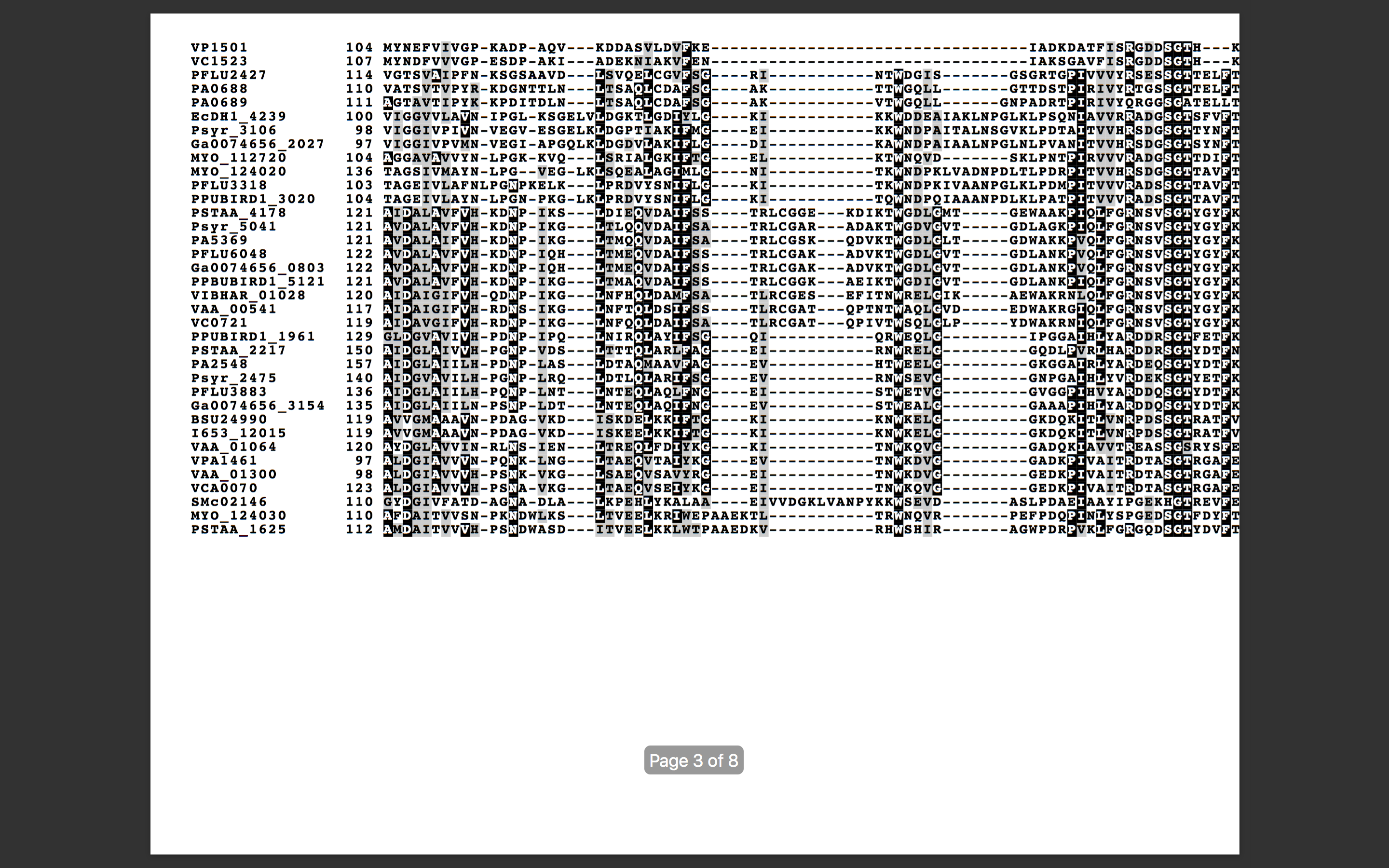


**Figure S3.** Conservation of the key residues (highlighted in red) involved in phosphate binding among the periplasmic binding proteins containing the domain, Pfam12849- PBP. Locus tags are used as the identifier. Abbreviations: VP, *Vibrio parahaemolyticus*; VC/VCA, *V. cholerae*; VAA, *V. anguillarum*; *V. harveyi* MYO, *Synechocystis* sp. PCC6803; PFLU, *P. fluorescens*; PA, *P. aeruginosa*; PPUBIRD1, *P. putida*; PSTAA, *P. stutzeri*; Psyr, *P. syringae*; EcDH1, *E. coli*; *P. Antarctica*; Smc; *Ensifer meliloti*.

WT_BIRD1

*Δ*_BIRD1

DSM4166

SBW25

PhnD5

PhnD3

PhnD

PhnD 2

PhnD4

PhnD3

PhnD2

PhnD4

PhnD3

PhnD2

**Figure S4.** Semi-quantitative abundance analysis of the putative phosphonate substrate binding proteins detected in the exoproteomes of the three *Pseudomonas* strains. and *the phoBR* mutant. Results presented are the mean of triplicate cultures. Error bars denote standard deviation.

A)

B)

**Figure S5** Growth of the *phoBR* mutant strain of *P. putida* BIRD-1. **(A)** A comparison of the *phoBR* mutant grown under Pi-replete (Black circles) and Pi-deplete (Grey circles) conditions. Concentrations of Pi were the same as those used for the wild type. Black arrows indicated the times of sampling for proteomics and exoproteomics. The striped arrow indicates the addition of Pi (50 μM) to help generate enough biomass for sampling. **(B)** Growth yields of either the wild type or *phoBR* mutant sampled after 48 hours grown on Pi-replete or Pi-deplete growth media. Results presented are the mean of triplicate cultures. Error bars denote standard deviation.

Figure S6. Figure. Evolutionary relationships of PhoX-like homologs. The evolutionary history was inferred using the Neighbor-Joining method [1]. The optimal tree with the sum of branch length = 3.66263884 is shown. The tree is drawn to scale, with branch lengths in the same units as those of the evolutionary distances used to infer the phylogenetic tree. The evolutionary distances were computed using the p-distance method [2] and are in the units of the number of amino acid differences per site. The analysis involved 27 amino acid sequences. All ambiguous positions were removed for each sequence pair. There were a total of 842 positions in the final dataset. Evolutionary analyses were conducted in MEGA6 [3].

References

1. Saitou N. and Nei M. (1987). The neighbor-joining method: A new method for reconstructing phylogenetic trees. Molecular Biology and Evolution 4:406-425.

2. Nei M. and Kumar S. (2000). Molecular Evolution and Phylogenetics. Oxford University Press, New York.

3. Tamura K., Stecher G., Peterson D., Filipski A., and Kumar S. (2013). MEGA6: Molecular Evolutionary Genetics Analysis version 6.0. Molecular Biology and Evolution30: 2725-2729.

**Supplementary datasets**

Table S1: A rank-abundance profile of the identified proteins in the exoproteome of *Pseudomonas putida* BIRD-1.

Table S2: A rank-abundance profile of the identified proteins in the exoproteome of *Pseudomonas fluorescens* SBW25

Table S3: A rank-abundance profile of the identified proteins in the exoproteome of *Pseudomonas stutzeri* DSM4166

Table S4: A rank-abundance profile of the identified proteins in the proteome of *P. putida* BIRD-1

Table S5: A rank-abundance profile of the identified proteins in the cellular proteome of the *P. putida* BIRD-1 *phoBR* mutant

Table S6 A rank-abundance profile of the identified proteins in the exoproteome of the *P. putida* BIRD-1 *phoBR* mutant
